# Efficient spectral data reduction for accurate iodine quantification in multi-energy CT

**DOI:** 10.1038/s41598-025-11270-w

**Published:** 2025-07-18

**Authors:** Olivia F. Sandvold, Roland Proksa, Heiner Daerr, Amy E. Perkins, Kevin M. Brown, Thomas Koehler, Ravindra M. Manjeshwar, Peter B. Noël

**Affiliations:** 1 Department of Radiology, Perelman School of Medicine, University of Pennsylvania, Philadelphia, USA; 2 Department of Bioengineering, University of Pennsylvania, Philadelphia, USA; 3 Philips Innovative Technologies, Hamburg, Germany; 4 Philips Healthcare, Cleveland, USA

**Keywords:** Computed tomography (CT), Quantitative imaging, Spectral CT, DECT, kVp-switching, Iodine contrast, Multi-energy CT, X-ray tomography, Imaging techniques

## Abstract

This study proposes a spectral data reduction method for multi-channel computed tomography (CT) that optimizes material decomposition accuracy while minimizing data complexity. Spectral CT enables quantitative assessments by utilizing multiple spectral channels, yet the associated noise and computational demands can limit its clinical application. We introduce a weighting scheme that reduces acquired four spectral channels—derived from a dual-layer, rapid kVp-switching (kVp-S) CT setup—into two optimized input channels for material decomposition. This scheme minimizes noise in iodine and water decomposition tasks by optimizing weights based on the Cramer-Rao lower bound. We modeled various duty cycles and patient sizes and compared results to full four-channel and traditional kVp-S configurations. The two-input weighting schemes showed consistently low estimated noise performance within 0.27% difference to the ideal, four-input material decomposition results for all tested duty cycles in a standard adult-sized 300 mm water phantom. In the pediatric (150 mm) and large adult (400 mm) phantom cases, the two-input weighted schemes were within 1% difference of the ideal four-input noise estimator results on average across all tested duty cycles. This study shows that optimized two-channel weighting in spectral CT matches the accuracy of four-channel setups for material decomposition, reducing noise and computational demands.

## Introduction

Spectral computed tomography (CT) enables quantitative imaging results, providing physiologic and functional insights to enhance CT diagnostic utility^1,2^. Additional spectral results including monoenergetic images and material-specific maps allow for increased lesion detection, decreased noise, and advanced tissue analysis^3–6^. From a technological standpoint, there are several approaches to enable clinical spectral CT^7,8^. These include: dual-source geometry^9^, slow/spin–spin and rapid kVp-switching (kVp-S)^10,11^, spectral detectors (energy-integrating or photon-counting)^12,13^, and twin-beam filters^14^. Physics-based constraints exist for each of these implementations, particularly relating to spectral separation^15^, susceptibility to motion artifacts^16^, electronic noise and scatter^17^, and spectrum modeling mismatch^18^, all of which generate noise and produce bias. In ultra-low dose scenarios, heightened noise levels may further increase bias in spectral results^19^. These limitations are a challenge for quantitative tasks such as measuring iodine uptake in oncological lesions where high sensitivity of iodine estimation is required to evaluate therapy response and where the correct identification of metastases is dependent on accurate CT characterization^6,20^.

Prior work has demonstrated that CT systems designed to produce more than two spectral datasets can yield lower bias and lower noise, particularly for low-concentration iodine^21–24^. One way to obtain additional spectral information is using a photon-counting detector (PCD) with three or more energy bins^25^. Another possibility is to use a hybrid spectral CT system combining dual-energy technologies. The ability to select a subset of the acquired photon energy information to maximize spectral diversity and minimize noisy input signals is a desirable trait of both hybrid and PCD technology. Several publications have shown that generating and using more than two energy-segregated data channels improves spectral performance and enables three-material decomposition^26–28^. Design optimization of such systems considering clinical processing time and resource limitations has yet to be further explored and implemented into a clinical workflow.

This study presents weighting schemes that reduce the number of energy bins required in the projection space for multi-bin CT acquisitions. This approach enables the application of computationally efficient, well-established two-input decomposition techniques. These two-input algorithms are widely adopted in clinical settings, where the majority of applications only require two-material decomposition, such as for water and iodine. By reducing the projection space data channels from four to two, we improve compatibility with existing clinical processing workflows, potentially reduce data transfer demands from the slip ring, and enable optimized selection for high-performance spectral imaging.

Material decomposition approaches in spectral CT determine the mass attenuation contributions from either the photoelectric effect and Compton scatter interactions or between two clinically relevant material basis functions, e.g., water and iodine, to estimate underlying tissue properties^1,29^. The Cramer-Rao lower bound of variance (CRLB) has been demonstrated for the optimization of design parameters and estimation of the quantitative performance of unique CT system configurations^29–31^. The CRLB predicts the minimum achievable noise correlation based on quantum statistics, detector imperfections, and realistic tube spectra provided in the simulation. It excludes analysis of patient-based artifacts such as motion or improper positioning. Direct modeling of bias is not possible with the CRLB as the process assumes an unbiased estimator. One advantage of this statistical approach is that the CRLB can be readily extended to include more than two input spectra and two output basis material estimations. However, in a clinical CT system, increasing the number of spectral inputs or desired material decompositions is complex and increases computational burden. Therefore, we realistically constrain our material decomposition schemes to use only two spectral inputs composed from weighted sums of the multiple data channels acquired and to estimate water and iodine noise in a simplified phantom. Each data channel’s optimal contribution was determined by minimizing the expected CRLB noise.

The foundation of this proposed weighting methodology builds upon the knowledge that excellent spectral separation is essential for high-accuracy material quantification in spectral CT^1^. In photon-counting technology, spectral separation is a product of photon energy thresholding to create multiple energy bins. In comparison, the theorized hybrid CT configuration considered here includes a dual-layer detector and kVp-S tube, which would produce four distinct “data channels” of energy-integrated signals provided by combinations of high and low tube voltages with upper and lower detector layers. The spectral separation would arise from the discrimination of two distinct polychromatic spectra by the dual-layer detector. To both feasibly handle multi-channel data and optimize the addition of the data to a general spectral forward model, we introduce a weighting method to do the following: (1) implement a linear weighting scheme to combine the four channels into two new spectral inputs; (2) use the CRLB as an optimization function to identify optimal weights over varying scan parameters. We use a simulation to compare the noise estimate from our simplified two-channel weighting schemes to an idealized four-channel performance. In this paper, we show how using these weighting schemes to compress spectral data achieves noise statistics that are on par with full spectrum four-channel noise estimates.

## Methods and materials

### Phantom materials

In this experiment, a simulated phantom was modeled by defining path lengths through water [1 g/mL] and iodine [10 mg/mL]. For an ‘average’ adult-sized patient, the path length was set to 300 mm of water to model a patient diameter of 300 mm. This corresponded to average waist circumferences of the 50th percentile of female adults and between the 25th and 50th percentile of adult males in the United States^32^. Our large adult patient was modeled with a 400 mm water path length to match the 90th percentile of adult males and between the 90th and 95th percentile of adult females^32^. Finally, a pediatric diameter of 150 mm was used to roughly approximate the 50th percentile of 2-year-old male and female children in the United States^33^. All iodine path lengths were set to 25 mm to represent a small lesion or vessel. Mass attenuation values were provided by the National Institute of Standards and Technology (NIST)^34^.

### Scan parameters

The hybrid spectral CT system applied in this study includes a rapid kVp-S source and a dual-layer detector. A realistic rapid kVp-S tube emission was modeled from a commercial system (vMRC 800, Philips Healthcare) operated in rapid switching mode between 140 and 80 kVp using manufacturer-specified spectra estimating the number of photons/keV/mA/s/sr defined from 10 to 150 keV in 1 keV intervals^35,36^. These spectra were generated using SpekCalc software^37–39^. Attenuation from tube housing, aluminum filtration, and a commercial bowtie filter were applied using known attenuation values from NIST. The tube current was set based on patient diameter. For the 300 mm, or average adult-sized phantom setting, a current of 200 mA was used for the 140 kVp projection, and 146 mA was used for the 80 kVp projection. The current difference accounts for the manufacturer’s expected decrease in emission current for ultrafast switching to lower tube voltage. Although the emitter temperature of the x-ray tube stays constant, the field-related emission drops. Table 1 shows the tube current used for each tube voltage for the pediatric and large adult phantoms. The rotation time was two seconds in total, with 2,000 projections per tube voltage.

**Table 1:** Simulation parameters.

Simulation parameter	High kVp	Low kVp
Tube voltage (kVp)	140	80
Tube current (mA)		
150 mm phantom	100	73
300 mm phantom	200	146
400 mm phantom	300	219
Cycle time %	10	90
	25	75
	50	50
	75	25
	90	10

The central ray of the tube spectra for each tube voltage, represented as ${{\varphi }}_{\text{kVp}}\left(E\right)$ in Eq. 1, were each multiplied by detector layer responses, ${D}_{\text{layer}}\left(E\right)$, for the upper and lower scintillators. This produced four corresponding data channels, ${S}_{\text{kVp,layer}}\left(E\right)$. The dual-layer detector responses contained the spectral sensitivities of the two detector layers (a ytterbium-garnet upper layer and gadolinium oxysulphide lower layer scintillator) with a centrally positioned geometry^35^. The manufacturer supplied these curves, including detector imperfections such as K-escape and layer crosstalk. Note that the upper layer scintillator signal was mainly composed of low energy photon signal while the lower layer signal was primarily composed of response to high energy photons. Cycle time, or time spent in one high and low kVp cycle, was kept constant at 1 ms. The duty cycle, or ratio of percent of cycle time spent using 140 kVp tube voltage compared to 80 kVp in one pair of projections, was initially set to 50/50.

1 $$\begin{array}{*{20}c} {S_{{\text{kVp,layer}}} \left( E \right) = \varphi_{{{\text{kVp}}}} \left( E \right) \cdot D_{{{\text{layer}}}} \left( E \right)} \\ \end{array}$$

$${\text{kVp}} \in \left\{ {{8}0,{ 14}0} \right\},{\text{ layer}} \in \left\{ {{\text{lowE}},{\text{ highE}}} \right\}$$

An estimated patient radiation dose per sr was given by $\sum_{\text{kVp}}^{\left\{140, 80\right\}}{\int }_{10}^{150 {\text{keV}}}{{\varphi }}_{\text{kVp}}\left(E\right)\cdot E dE$, or the sum of estimated photon energy in one pair of high and low kVp incident spectra. We referred to this value as d_140+80,_ while d_140_ and d_80_ represented the estimated sum of photon energy for the 140 and 80 kVp components, respectively. d_140+80_ was proportional to the airkerma for the scan. We remodeled the spectra ${{\varphi }}_{\text{kVp}}\left(E\right)$ 12 times, using varying duty cycles ranging from 0.1/99.9 to 90/10, including 10/90, 25/75, and 75/25, keeping total cycle time of 1 ms. A new estimated patient radiation dose was calculated for each new duty cycle ratio. We linearly scaled the new spectra to contain an equivalent total photon energy sum, d_140+80_, to the initial 50/50 duty cycle. Therefore, we created a single pre-patient “dose” level across duty cycles equal to the patient radiation dose per sr in the 50/50 case.

### Weighting scheme design

We used a two-input CRLB model for the two-channel material decomposition approach. To do this, we defined two new spectra using weighted combinations of the available four data channels. The weights, ${W}_{\text{kVp, layer}}$, for each channel indicated the contribution of those channel spectra to the final input decomposition channels (*i*) (Eq. 2). Summing over the linearly weighted channel spectra resulted in two new energy spectra, ${\Phi }_{i}\left(E\right)$, one for each decomposition input (Eq. 3).

2 $$\begin{array}{*{20}c} \begin{aligned} \overline{W}_{i} = & \left[ {W_{80, lowE} , W_{80, highE} , W_{140, lowE} , W_{140, highE} } \right] \\ {\text{i}} = & {1},{ 2} \\ \end{aligned} \\ \end{array}$$

3 $$\begin{array}{*{20}c} \begin{aligned} {\Phi }_{i} \left( E \right) = & \mathop \sum \limits_{{\text{kVp, layer}}}^{{}} S_{{\text{kVp,layer}}} \left( E \right) \cdot \overline{W}_{{i,{\text{kVp,layer}}}} \\ {\text{i = }} & {\text{1, 2, kVp }} \in { }\left\{ {80,{ }140} \right\},{\text{ layer }} \in { }\left\{ {{\text{lowE}},{\text{ highE}}} \right\} \\ \end{aligned} \\ \end{array}$$

**Fig. 1:**
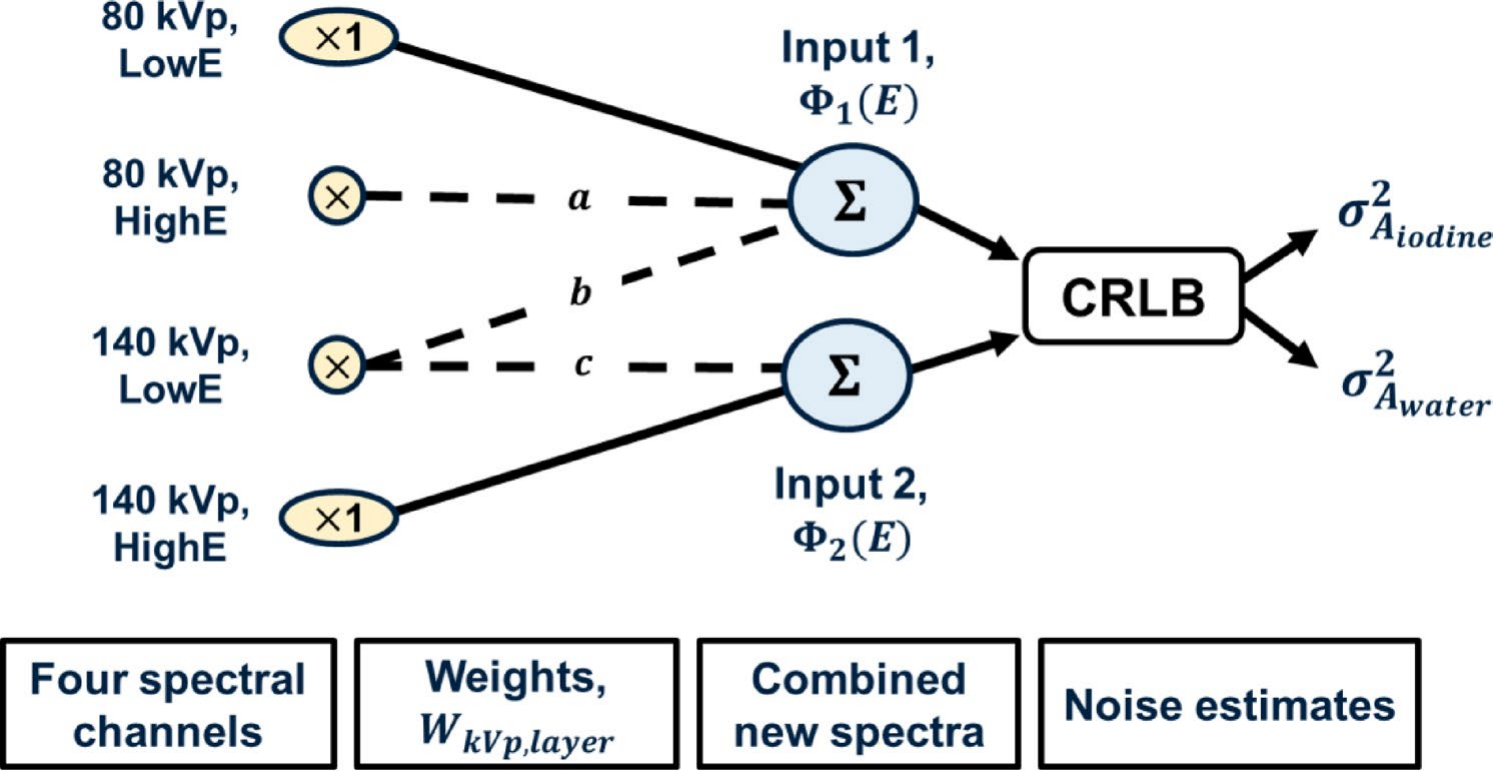
Outline of the methodology to reduce complexity of input dimensions through the linear combination of four channel inputs into two new spectra. The CRLB uses these two inputs (Φ_1_ (E), Φ_2_ (E)) based on weighting scheme and the weights (a, b, c) to estimate the noise in the projections for two basis materials, iodine and water. The contribution weights for the 80 kVp, low energy channel and 140 kVp, high energy channel have values of 1.

Supposing that there existed two potential states (zero or non-zero) for a single weight value, of which there were four in each weight matrix, ${\overline{W} }_{i}$ and using two weight matrices, a total of 2^8^ possible combinations could have been implemented with this method. However, this number neglects the removal of impractical zero-weight matrices. Realistically, the number of reduced dimension weighting schemes remained within single digits. In this paper, two general two-input channel schemes were proposed (Fig. 1) to simplify the number of possible optimization points. We excluded other combinations as they showed inferior noise performance. For both schemes, the *a* weight determined the contribution of high energy signals from the 80 kVp projection to the first material decomposition input to the CRLB. In the first scheme, the contribution of the low energy signals from the 140 kVp projection (denoted by *b*) was combined with a mix of the dual-layer detector signals from the 80 kVp projection to form one new low energy CRLB decomposition input. This mix comprised 100% low energy 80 kVp signals plus *a* weighted high energy 80 kVp signals. The high energy signals in the 140 kVp projection served alone as the second CRLB input. This contrasted with the second scheme where the low energy signals in the 140 kVp projection (*c* weighted) were added to the 140 kVp, high energy signals as the second CRLB input; the first CRLB input consisted of only 80 kVp signals with optimized weight *a*. Using defined weight notations, the weighted schemes were as follows:

$$\begin{aligned} {\text{Scheme 1}}: \overline{W}_{1} = & \, \left[ {{1},a,b, \, 0} \right] \, \;{\text{and}}\;\overline{W}_{2} = \, \left[ {0, \, 0, \, 0,{ 1}} \right] \\ {\text{Scheme 2}}: \overline{W}_{1} = & \, \left[ {{1},a, \, 0, \, 0} \right] \, \;{\text{and}}\;\overline{W}_{2} = \, \left[ {0, \, 0,c,{ 1}} \right] \\ \end{aligned}$$

where kVp-S was modeled by setting *a* = *1, b* = *0, c* = *1* or ${\overline{W} }_{1}$ = [1, 1, 0, 0] and ${\overline{W} }_{2}$ = [0, 0, 1, 1].

We generated one additional, final dimensionally reduced weighting strategy by taking the minimal number of channels (2) using only the maximally spectrally separated data channels (*a, b, c* = *0*) or ${\overline{W} }_{1}$ = [1, 0, 0, 0] and ${\overline{W} }_{2}$ = [0, 0, 0, 1]. While the dose efficiency of this combination was low, it was instructive to explore the impact of spectral separation versus photon fluence on material decomposition performance using this method compared to kVp-S and weighting schemes 1 and 2.

### Performance statistics

Spectral CT reconstructions attempt to recover line integrals, A_k_, for each projection, where ${a}_{k}\left(x,y\right)$ denote the k^th^ basis material density at each physical location (Eq. 4). In this paper, we utilized the CRLB to estimate the noise on an iodine/water decomposition task through a simple phantom where k represented iodine or water (Eq. 5).

4 $$\begin{array}{*{20}c} {A_{k} = \int {a_{k} \left( {x,y} \right)} ds} \\ \end{array}$$

5 $$\begin{array}{*{20}c} {\int {\mu \left( {x,y;E} \right) } ds = A_{{{\text{iodine}}}} \cdot \mu_{{{\text{iodine}}}} \left( E \right) + A_{{{\text{water}}}} \cdot \mu_{{{\text{water}}}} \left( E \right)} \\ \end{array}$$

We derived the CRLB to compare a four-channel estimator performance to simplified weighted two-channel estimators. In the four-channel estimation of noise, the four spectra ${S}_{\text{kVp,layer}}\left(E\right)$ were assumed to be independent signals. In the dimensionally reduced approach, new weighted spectra ${\Phi }_{1}\left(E\right)$ and ${\Phi }_{2}\left(E\right)$ were considered independent signals. For simplicity and to generalize both estimators, we referred to all these spectra as *S*(*E*) in the following equations. We assumed Gaussian noise on each measurement, ${m}_{j}$, the ${j}^{th}$ total energy deposited in $j=1,\dots , N$ total photon integrating measurements. Assuming all $N$ measurements were statistically independent, we defined the mean and variance of ${m}_{j}$ as $\theta$ and ${\sigma }^{2}$ respectively (Eq. 6–7). In the two-channel scenarios, we scaled the variance of the input signals ${\Phi }_{i}\left(E\right)$ with respect to the square of each element in ${\overline{W} }_{i}$ (Eq. 7). This ensured that the modeled noise reflected the relative contribution of each data channel to the final signal.

6 $$\begin{array}{*{20}c} {\theta = \int {E \cdot S} \left( E \right) \cdot \exp \left[ {\mathop \sum \limits_{{\begin{array}{*{20}c} {k \in {\text{\{ iodine,}}} \\ {{\text{water\} }}} \\ \end{array} }} - A_{k} \mu_{k} \left( E \right)} \right]dE } \\ \end{array}$$

7 $$\begin{array}{*{20}c} {\sigma_{{}}^{2} = \int {E^{2} \cdot \exp } \left[ {\mathop \sum \limits_{{\begin{array}{*{20}c} {k \in {\text{\{ iodine,}}} \\ {{\text{water\} }}} \\ \end{array} }} - A_{k} \mu_{k} \left( E \right)} \right] \cdot \mathop \sum \limits_{{\text{kVp, layer}}}^{{}} S\left( E \right) \cdot \left( {\overline{W}_{{\text{kVp, layer}}} } \right)^{2} dE} \\ \end{array}$$

$${\text{kVp}} \in \left\{ {80,{ }140} \right\}, {\text{layer}} \in \left\{ {{\text{lowE}},{\text{ highE}}} \right\}$$

The likelihood of obtaining the actual measurement, $m_{j}$, could be expressed as such (Eq. 8):

8 $$\begin{array}{*{20}c} {L\left( {m_{1} , \ldots ,m_{N} {|}\theta ,\sigma_{{}}^{2} } \right) = \mathop \prod \limits_{j = 1}^{N} \frac{1}{{\left( {2\pi \sigma_{{}}^{2} } \right)^{\frac{1}{2}} }} \cdot \exp \left[ {\frac{{ - \left( {m_{j} - \theta_{{}} } \right)^{2} }}{{2\sigma_{{}}^{2} }}} \right] } \\ \end{array}$$

The negative log-likelihood of (Eq. 9) then followed:

9 $$\begin{array}{*{20}c} {l\left( {m_{1} , \ldots ,m_{N} {|}\theta ,\sigma_{{}}^{2} } \right) = \frac{N}{2}\ln \left( {2\pi } \right) + \mathop \sum \limits_{j = 1}^{N} \left( {\frac{1}{2} \ln \left( {\sigma_{{}}^{2} } \right) + \frac{{\left( {m_{j} - \theta_{{}} } \right)^{2} }}{{2\sigma_{{}}^{2} }}} \right) } \\ \end{array}$$

Because the negative log-likelihood was dependent on ${A}_{\text{iodine}}$ and ${A}_{\text{water}}$, we computed the Fisher information matrix, $I\left(\theta \right)$, by taking the expectation of the partial derivative of all likelihood functions with respect to ${A}_{\text{iodine}}$ and ${A}_{\text{water}}$ (Eq. 10).

10 $$\begin{array}{*{20}c} {\left[ {I\left( \theta \right)} \right] = Expectation\left[ { - \frac{{\partial^{2} \ell }}{{\partial A_{{{\text{iodine}}}} \partial A_{{{\text{water}}}} }}} \right]} \\ \end{array}$$

The CRLB states for an unbiased estimator that the lower bound of variance, assuming it to be finite, is as follows (Eq. 11):

11 $$\begin{array}{*{20}c} {Var\left( \theta \right) \ge \frac{1}{I\left( \theta \right)}} \\ \end{array}$$

**Fig. 2:**
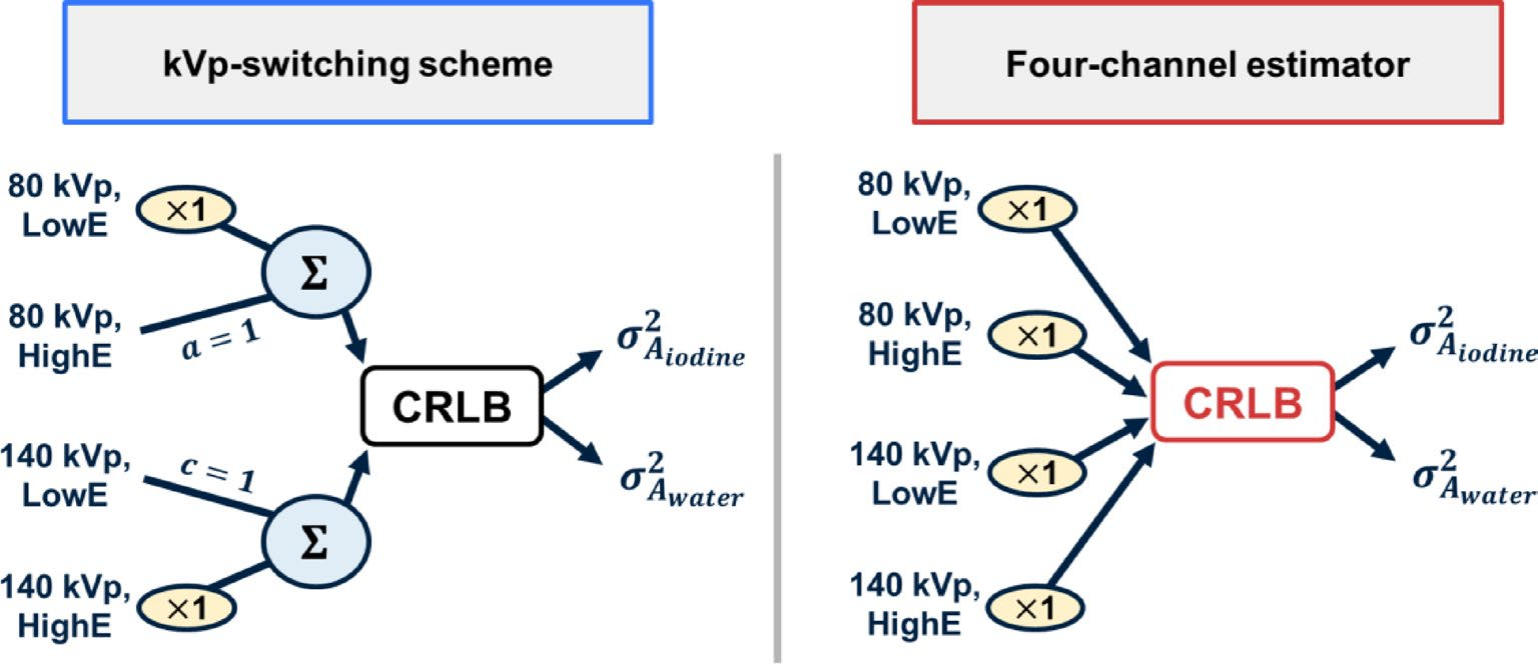
Graphical representation of the reduced dimension two-channel kVp-S estimator weighting scheme (left) and the four-channel estimator (right) used to compute the CRLB noise estimates. Both approaches use 100% of the available spectral channels, but the kVp-S scheme combines layer information before noise estimation.

In the pure kVp-S scenario, the input weight matrices $\overline{W }$ were [1, 1, 0, 0] and [0, 0, 1, 1] to represent the full utilization of the available 80 kVp information from both detector layers as one input to the decomposition and the full use of the 140 kVp in both layers as the second input. The visualization of this approach is shown in Fig. 2 on the left, with the four-channel CRLB estimator approach schematic on the right. The four-channel estimator provided a baseline performance metric against which to compare different weighting strategies. However, it is undesired to implement as a complete material decomposition method with real data due to computational complexity and possible additional burden on the slip ring. While both kVp-S and the four-channel estimators used the full spectrum available, the difference was whether to combine layer signals before calculating the spectral noise. We computed the CRLB estimate of iodine and water noise for four-channel and kVp-S for each duty cycle and phantom size combination.

### Weight optimization

To identify the values for *a, b* (scheme 1) and *a, c* (scheme 2) to minimize the CRLB noise estimates, we used the Python Scipy basin-hopping minimization algorithm, where the objective function was the iodine noise estimate. The basin-hopping approach iteratively attempts to find the global minimum of the described function using random perturbation of initialization weights alongside local minimization. The truncated Newton Conjugate-Gradient algorithm was used to locate the weight parameters at the minimum CRLB noise for each pairing of duty cycle and phantom size. An initialization weight of 0.5 was used with a maximum basin-hopping step size of 0.33 and total iteration number set to 2500. Valid weight values ranged from [0, 1]. No discontinuities or instabilities were identified when the initialization weights were systematically varied from 0 to 1. Convergence, with function minimization tolerance < 10^−6^, was obtainable for all combinations of duty cycle and phantom size.

## Results

### Effect of photon energy ratios on duty cycle and estimated noise

**Fig. 3:**
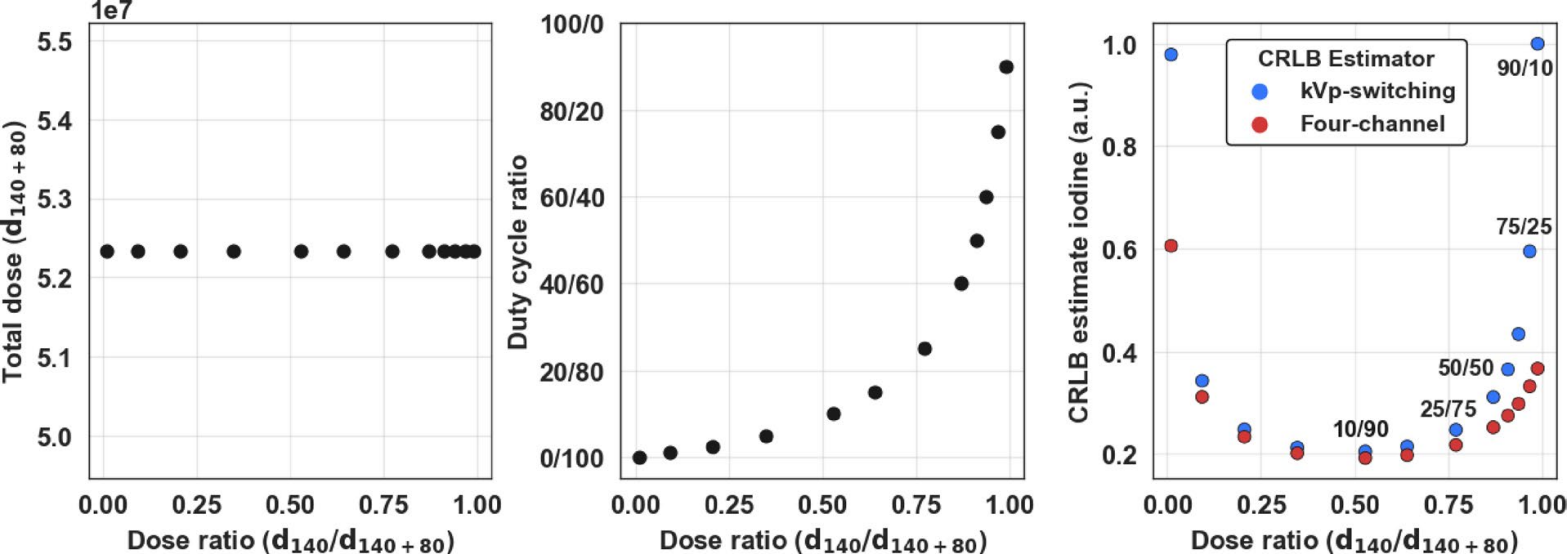
Demonstration of the effect of the ratio d_140_/d_140+80_, while d_140+80_ is held constant (left). The duty cycle experiences an exponential positive change as d_140_/d_140+80_ increases (center). Duty cycle ratios near zero or one demonstrate high estimated noise for kVp-S and four-channel estimator (right). A 0.5 dose ratio of d_140_/d_140+80_ roughly corresponds to a 10/90 duty cycle.

We took a collection of 12 simulations using 300 mm of water and 25 mm of iodine with varying duty cycles from 0.1/99.9 to 90/10, including five selected duty cycles (10/90, 25/75, 50/50, 75/25, 90/10), and normalized the tube spectra to contain the same total photon energy as a 50/50 duty cycle acquisition. Figure 3 illustrates the relationship between the fraction of high kVp photon energy (d_140_) relative to the total photon energy (d_140+80_) with the corresponding duty cycles and estimated relative CRLB iodine noise using kVp-S (*a* = *1, b* = *0, c* = *1*) and the four-channel estimator. The CRLB noise was normalized to the maximum noise produced by the kVp-S acquisition. The 10/90 duty cycle was closest to containing equivalent spectra photon energy between 140 and 80 kVp projections with the selected scan parameters. At this duty cycle ratio, kVp-S and four-channel CRLB estimates were at their minimum. Extreme duty cycle ratios in either direction resulted in high estimated noise as the distribution of SNR between the two voltages’ spectra became highly unbalanced. Comparing kVp-S and four-channel noise estimates at different duty cycle ratios, we noted that four-channel noise was always lower and more advantageous, especially at extreme duty cycle ratios. For example, using the 90/10 duty cycle, the four-channel iodine noise was nearly one-third of the predicted kVp-S iodine noise. This demonstrates the additional spectral separation capabilities of the four-channel detection. The comparison highlights the potential benefit a dual-layer detector may add to a kVp-S system.

### Effect of duty cycle and weight values on CRLB noise estimate

**Fig. 4:**
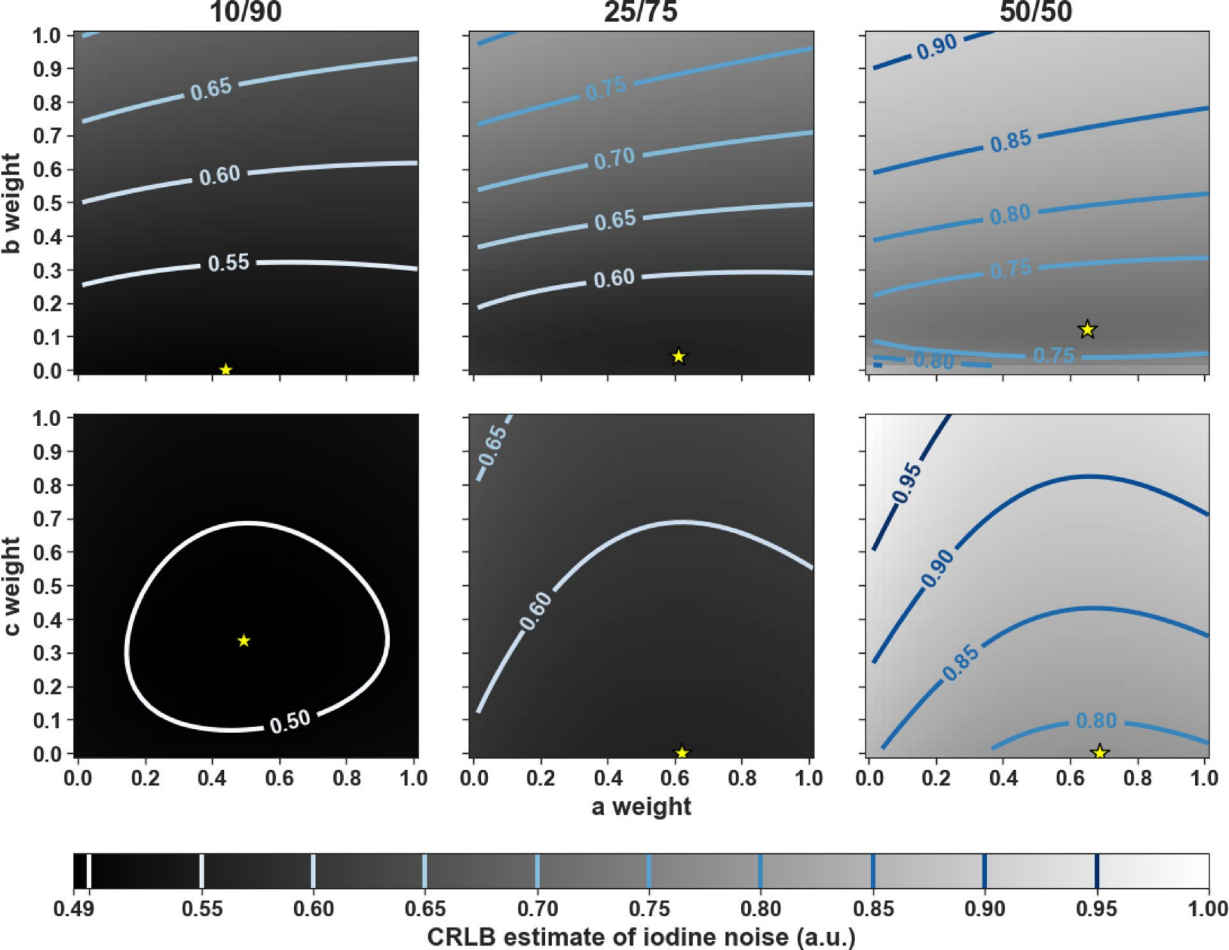
Using the 300 mm adult water length, we show the relationship comparing scheme 1 (upper row) and scheme 2 (lower row) weight values on estimated CRLB iodine noise relative to the max noise estimate in the 50/50 dataset. The yellow stars indicate the weights with the lowest iodine noise estimate. In the 10/90 and 25/75 duty cycles, scheme 2 outperformed scheme 1; however, scheme 1 had the lowest iodine noise estimate in the 50/50, 75/25, and 90/10 duty cycles. Average noise estimates increased as the duty cycle included an increased 140 kVp cycle time.

Using 40 weights linearly spaced from 0 to 1 for each input weight, the CRLB estimation of iodine noise in the same 300 mm water phantom was plotted to examine the relationship between duty cycle, weighting scheme 1, and scheme 2. The noise values were normalized to the maximum CRLB output between both schemes using the 50/50 duty cycle as a reference to visualize a uniform scale across the 10/90, 25/75, and 50/50 plots (Fig. 4). The optimal weights producing the lowest noise were marked with a yellow star. Figure 4 shows the clear impact of the duty cycle: as the 140 kVp component increased, the average normalized noise value in each plot increased, going from 0.590 to 0.667 to 0.808, and 0.504 to 0.598 to 0.876 for schemes 1 and 2, respectively. Scheme 2 had a lower minimum CRLB iodine noise in the 10/90 and 25/75 duty cycles, whereas scheme 1 generated the lowest expected iodine noise in the 50/50, 75/25, and 90/10 duty cycles. Across all duty cycles, the optimal weight for *a* ranged from 0.429 to 0.675, while all optimal *b* and *c* weights remained less than or equal to 0.153 and 0.327, respectively. In duty cycles 25/75 and higher, the *c* weight to optimize the CRLB iodine noise was 0. In both schemes, as the 140 kVp component of the duty cycle increased, the optimal *a* value also increased.

### Comparison of two-channel weighting schemes to the four-channel case with respect to noise estimate

**Fig. 5:**
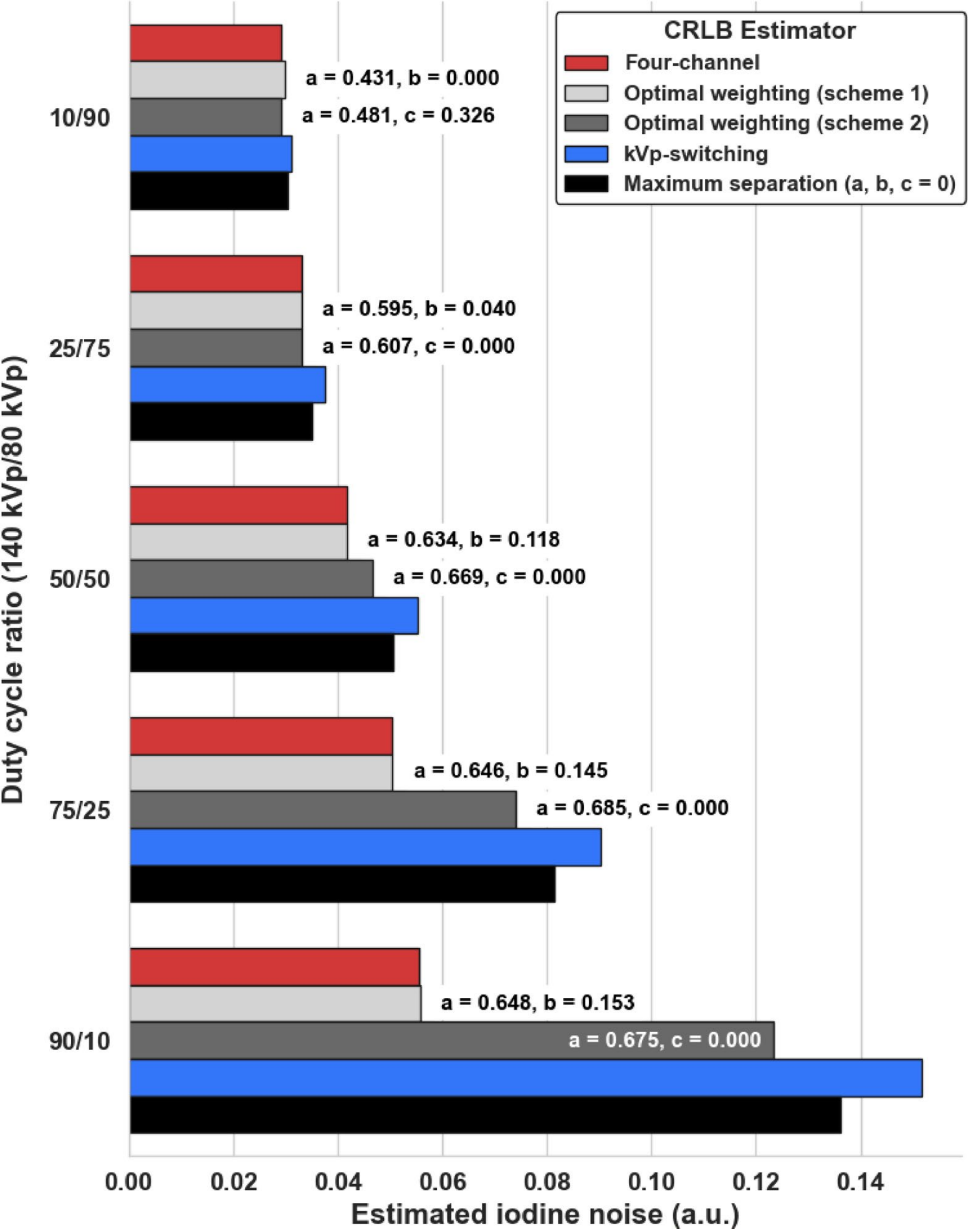
Comparison of two-channel CRLB weighted schemes relative to the four-channel estimator iodine noise for each duty cycle for the 300 mm patient size. As the 140 kVp component of the duty cycle increases, the noise estimates for kVp-S noise and maximally separated weighting become larger. In comparison, the optimal weighting scheme, particularly scheme 1, remains robust and capable of achieving noise levels within 0.26% of the four-channel estimate, given by proximity to the red bar, regardless of duty cycle.

The estimated iodine noise using the reduced dimension two-channel estimator schemes compared to the four-channel estimator were visualized for the 300 mm patient diameter (Fig. 5). The bar in red represented the four-channel, ideal material decomposition noise performance given the tube spectra, detector response, and spectral separation at each duty cycle. This scheme contained the lowest noise across all tested schemes for all duty cycles and patient thicknesses. As the 140 kVp component increased, the estimated noise for all schemes increased, although the magnitude varied for each scheme. From the 10/90 duty cycle to the 90/10 duty cycle, the four-channel noise nearly doubled while the kVp-S noise more than tripled. The optimal weighting for scheme 2, kVp-S, and the maximally separated weighted noise estimates were over 2x the estimated four-channel noise using the least favorable duty cycle, 90/10. In contrast, the optimal weighting scheme 1 noise was only 1.0026x greater than the four-channel estimate. The maximally separated reconstruction scheme CRLB estimate was always less than the kVp-S noise and greater than the optimal weighting schemes 1 and 2. These results importantly demonstrated that one of the two-channel weighting schemes, using optimal weights, produced an estimated iodine noise no greater than 0.27% of the four-channel noise. Scheme 2 performed better than scheme 1 only in the 10/90 duty cycle.

### Pediatric- and large adult-sized phantom weighting

For the 400 mm large adult case, we found the best two-channel performance was the optimal weighting scheme 2 in the 10/90 duty cycle, where the noise was only 0.05% greater than the corresponding four-channel noise. In the 90/10 duty cycle scenario, the best two-channel noise performance was scheme 1, with 1.015x the noise of the four-channel iodine noise. On average, kVp-S CRLB noise values were 1.536x greater than the ideal noise, while scheme 1 noise was only 1.01x greater than the lowest noise estimates. Trends for the 150 mm pediatric-sized phantom case were the same as observed for the other two phantom sizes. On average, the optimal weighted scheme 1 iodine noise was 1.006x greater than the four-channel estimates for the same duty cycles. In comparing these different water path lengths, we demonstrated that the weighting system could receive varying patient parameters and generate optimal weights to produce noise estimates similar in magnitude to the ideal four-channel material decomposition estimator.

## Discussion

We introduced a method to address data dimension reduction necessary for multi-channel spectral CT systems in clinically relevant two-material decomposition tasks. It is important to note that most current clinical applications only require two-material decomposition due to the absence of a K-edge material. Our ultimate goal for such a system is to maximally reduce bias in material decomposition, particularly for iodine contrast, because accurate iodine quantification and visualization increases the screening and diagnostic utility of CT imaging. These simulation studies can be easily modified to fit more diverse body sizes, iodine concentrations, and system parameters including tube spectra and detector sensitivities. This flexibility allows for a vendor-independent application. Most importantly, we provide a feasible solution to optimally use available data and integrate outputs using fast and efficient material decomposition methods. We showed that the performance of these weighted reduced-dimension two-channel systems matches the performance of a four-channel estimator.

The optimal weights determined by minimizing CRLB iodine estimated noise reveal how to best utilize the additional spectra data by balancing photon fluence and the desirable photon energy separation. Given scheme 1 produced the lowest noise in all tested duty cycles except 10/90, we showed that 140 kVp, low energy photon information from the upper detector layer combined with the 80 kVp projections creates a more optimal spectral input rather than combining the layer data with the 140 kVp when switching parameters favor the 140 kVp tube voltage component. We replicated these findings for multiple simulated patient sizes. In practice, the scout or surview x-ray scan may be used to estimate patient size and selection of optimal weights for iodine/water material decomposition.

The use of the CRLB to predict the performance of material decomposition from spectral inputs has been described by Roessl and Hermann^30^ and Yang et al.^31^ using both dual-layer and photon-counting detector technology. Though we do not model every system imperfection (K-edge escape, electronic noise, scatter, noise-induced bias, anti-correlated noise, etc.), we used a precise physical model that captures primary spectral effects that directly play a role in material decomposition. In future studies with available CT instrumentations, we will apply these weights to fully integrate non-linear imaging chain effects which were not captured in this simplified simulation. Sensitivity to material decomposition inputs will be measured using qualitative image quality metrics and quantitative spectral performance. Observation of contrast-to-noise ratio (CNR) and detectability of small, low-contrast lesions will be quantified and compared between non-hybrid and hybrid, weighted channel images. These findings will provide increased clinical significance of the proposed method. One disadvantage of this experiment is the absence of a more complex simulated phantom. Physical experiments acquiring projections through phantoms of varying unknown sizes will further guide statistical analysis of the number of optimal weights that should be implemented for a given type of CT protocol. We will also explore optimizing channel reduction in three material decomposition tasks to take advantage of available multi-channel CT data and extend the presented weighting approach. While we selected one iodine concentration and only five duty cycles for testing hybrid kVp-S and dual-layer acquisitions, we recognize that additional clinical input will be required to define realistic protocols.

Future experimental translation will require overcoming several outstanding hurdles. First, access to a photon-counting or hybrid spectral-CT scanner is essential for conducting physical experiments and validating the proposed weighting scheme. Second, the optimization space is extensive—encompassing duty-cycle ratio, patient habitus, tube-voltage pairings, tube-current settings, and the competing goals of reducing iodine noise, minimizing energy-dependent noise, and limiting bias. Exclusively minimizing iodine noise, for example, can induce unacceptable spectral bias in quantitative tasks; thus, trade-offs should be quantified with well-characterized tissue inserts and evaluated against clinical tolerance limits. Finally, discrepancies between simulated physics and real-world detector behavior may distort optimal weights; such spectral mismatches can be mitigated through system-specific calibration and refined response modeling.

Finally, we note that this process could be replicated for a CT with a photon-counting detector and other tube configurations. Yang et al. demonstrated an eight-bin compression strategy for a silicon-based photon-counting detector that capitalized on the CRLB to find ideal weighting combinations^40^. We showed our weighting method using a theorized rapid kVp-S x-ray tube and dual-layer detector hybrid system. This configuration leverages the inherent high spectral separation of incident spectra while further discriminating the high kV and low kV acquisitions into low energy and high energy photon domains produced from the dual-layer detector. Experimental work acquired on clinical analog systems will be required to validate this method for managing and optimizing multi-channel spectral data.

## Conclusion

In this paper, we present a strategy for optimal dimensionality reduction of spectral data from multi-channel spectral CT systems by utilizing an analytical statistical approach. The results offer several significant advantages for CT imaging: (1) using established two-material decompositions provides a robust, extensively validated solution; (2) our approach supports the low noise and minimal bias required for clinical applications involving multi-bin acquisition with two-material decomposition; (3) computational complexity remains low, meeting clinical demands for data generation and reconstruction; and (4) applying this reduction directly to gantry data significantly decreases data transfer requirements over the slip ring, which is particularly beneficial for high-resolution spectral data. This latter benefit translates into reduced time and lower hardware costs.

In CT diagnostic imaging, the use of iodine contrast is paramount for the visualization of vascular processes and pathologies. This task becomes exceedingly difficult at lower radiation doses or with smaller iodine volumes and concentrations, both of which are desirable for increased patient safety^41^. Thus, there is demand for a spectral CT system that can accommodate low-concentration iodine, low radiation dose exposure, and delivery of accurate iodine quantification. Combining existing technologies or using state-of-the-art hardware has been theorized to fulfill these requirements, but optimizing these systems to utilize increased spectral information, in a clinically feasible timeframe, has yet to be fully explored. We demonstrate a flexible method to incorporate multi-channel spectral information in an optimized weighting configuration to produce ideal iodine quantification results across diverse patient sizes.

## Data Availability

Data generated during this study are available from the corresponding author upon reasonable request.
